# The impact of endometriosis on depressive and anxiety symptoms and quality of life: a systematic review

**DOI:** 10.3389/fpubh.2023.1230303

**Published:** 2023-09-06

**Authors:** Małgorzata Szypłowska, Rafał Tarkowski, Krzysztof Kułak

**Affiliations:** Chair and Department of Oncological Gynecology and Gynecology, Medical University of Lublin, Lublin, Poland

**Keywords:** women’s mental health, women’s psychological functioning, endometriosis, depression, anxiety, quality of life

## Abstract

**Introduction:**

Endometriosis is a common gynecological disorder affecting approximately 10–15% of women of reproductive age. The main complaints of patients with endometriosis are pain and fertility problems. Symptoms of endometriosis can impact the psychological functioning of the patients and significantly compromise their mental health.

**Methods:**

The aim of this review was to assess the prevalence of depressive and anxiety symptoms and quality of life in endometriosis patients. For this systematic review, we searched the PubMed, MEDLINE, ProQuest, EMBASE, Cochrane, CINAHL, Google Scholar, Scopus, and ScienceDirect electronic databases up to March 2023 to identify potentially relevant studies. The systematic review in the present paper is reported in accordance with the Preferred Reporting Items for Systematic Reviews and Meta-Analyses (PRISMA) guidance.

**Results:**

Of four records identified, 18 were eligible to be reviewed on the association between endometriosis and symptoms of depression and anxiety. Of 8,901 records identified, 28 were reviewed on the association between endometriosis and quality of life. The reviewed articles showed a prevalence ranging from 9.8 to 98.5% for depressive symptoms and 11.5 to 87.5% for anxiety. The quality of life in patients with endometriosis was significantly impaired, regardless of the tool used for evaluation.

**Discussion:**

This systematic review shows that endometriosis is associated with depressive and anxiety symptoms and impaired HRQoL. Broad correlating factors modulate mental health outcomes, indicating the complex relationship between the disease and the psychological health of the patients.

## 1. Introduction

Endometriosis is defined as the presence of endometrial cells outside their natural location. It is a common gynecological disorder that affects approximately 10–15% of women of reproductive age and up to 50% of women with pelvic pain and/or fertility problems (1, 2). Despite many years of research, the pathogenesis of endometriosis remains enigmatic. There have been many theories proposing the etiology of the disease, but no single one of them explains all the different clinical presentations and pathological features of endometriosis. It is possible that different subtypes of endometriosis may develop via different pathogenetic mechanisms (3). The main clinical manifestation of the disease is the presence of painful symptoms such as dysmenorrhea (pain during menstruation), dyspareunia (pain during sexual intercourse), chronic pelvic pain, acyclic pain, or dyschezia (painful defecation). The presence of pain affects the psychological and social functioning of endometriosis patients which has a profound impact on their quality of life. In endometriosis patients, the pain affects QoL more negatively than in women with other benign gynecological conditions. Moreover, each group is influenced by a different type of pain. The intensity of general perception of pain and dysmenorrhea is greater in women with endometriosis than those suffering from other painful diseases (4). Endometriosis significantly impacts the psychological functioning of the patients and compromises their mental health (5). Long-term history of endometriosis is associated with higher levels of perceived stress, suggesting that the chronicity of the disease is an independent factor affecting the perception of stress (6). Endometriosis is often present, and more often severe, among infertile women (7). Negative impact of the disease was also reported on obstetric outcomes. Women with endometriosis after natural conception have an increased risk of preterm delivery and neonatal admission to an intensive care unit, and when a severe adenomyosis is coexistent with endometriosis, a higher risk of placenta previa and cesarean delivery was observed (8).

The treatment of endometriosis includes surgical and pharmacological management. The choice of treatment method depends on the type and stage of the disease and the patient’s expectations. Therapy strategies aim mainly to increase QoL and improve fertility while lowering the risk of recurrence (9). Many medical and surgical treatments for endometriosis demonstrate comparable benefits, mainly in pain control and improvement in QoL (10). Surgical approach has a positive impact on organ impairment and sexual function in women with deep infiltrating endometriosis (DIE) (10) and shows a promising benefit on fertility outcomes (9). However, the repetitive surgery was revealed to have a potential negative role on psychological well-being of the patients, with an increasing number of interventions correlating with higher levels of perceived stress (6). Hormone therapy administered in the preoperative period can have a role in reducing endometriosis-associated pain; however, no significant differences on health-related quality of life were found (11).

## 2. Materials and methods

A systematic review was conducted to evaluate the prevalence of depressive and anxiety symptoms and quality of life assessment in patients with endometriosis. The systematic review in the present paper is reported in accordance with the Preferred Reporting Items for Systematic Reviews and Meta-Analyses (PRISMA) guidance (12).

### 2.1. Eligibility criteria

Participants with a diagnosis of endometriosis were included, regardless of the diagnostic examination performed. Included studies evaluated the occurrence of depressive and anxiety symptoms and assessed the quality of life in patients with endometriosis. Animal studies, case series and reports, reviews, published conference abstracts, and articles published in languages other than English or Polish were excluded. Articles with paid access to the full text were excluded.

### 2.2. Search strategy

A thorough literature search was conducted via PubMed (RRID: SCR_004846), MEDLINE (RRID: SCR 002185), ProQuest (RRID: SCR_006093), EMBASE (RRID: SCR_001650), Cochrane Central Register of Controlled Trials (RRID: SCR_006576), EBSCO CINAHL (RRID: SCR_022707), Google Scholar (RRID: SCR_008878), and Scopus (RRID: SCR_022559) electronic databases up to March 2023. The search strategy for reviewing the prevalence of depressive and anxiety symptoms included the use of the following Boolean operators: (“depressi*” OR “anxi*”) AND “endometriosis” in the titles and abstracts of articles. The sequence of terms used to identify the studies that evaluated the quality of life implemented the use of key terms such as (“quality of life” OR “QoL”) AND “endometriosis” in the titles and abstracts of the articles.

### 2.3. Selection process

Records identified through database searching were imported into a reference manager. All records were screened by title and abstract by two independent researchers, and potentially eligible studies were identified. The full texts were assessed against the inclusion criteria of the review by two independent reviewers. Any disagreements between screeners were solved by consensus, or the opinion of a third researcher was obtained. From each report included in the present paper, the data were extracted by one reviewer and checked by another. We collected data on variables of interest that included: the report (author, year of publication); number and characteristics of participants (sample size, mean age); the study (definition and criteria for endometriosis); and the main findings (how they were ascertained, what outcomes were assessed, other variables). When available, quantitative measurements of the outcomes assessed were collected, with means and standard deviations extracted as a first choice.

### 2.4. Selection

#### 2.4.1. Depressive and anxiety symptoms

**Figure 1:**
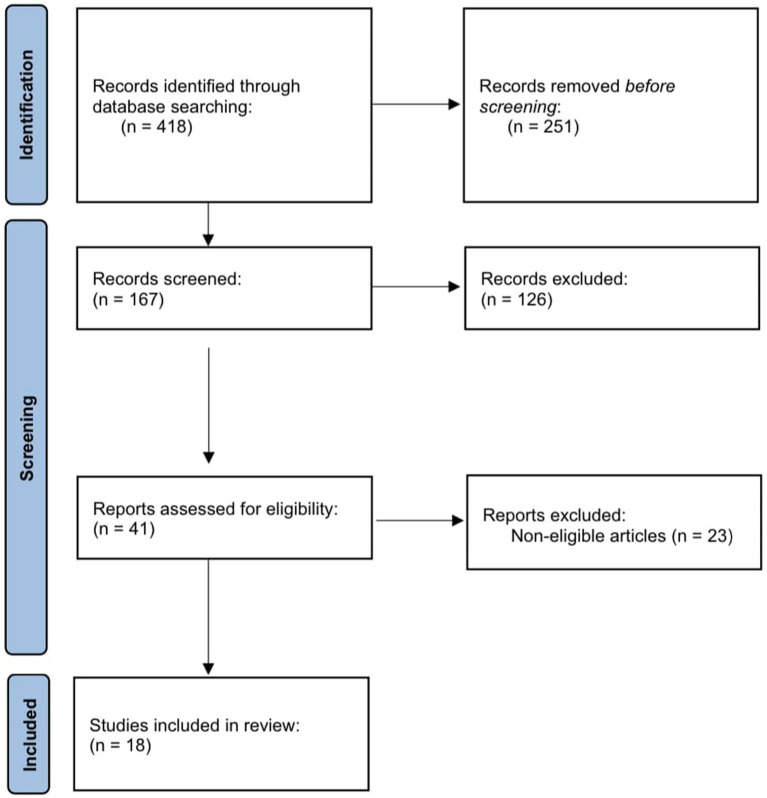
Flow diagram summarizing the selection process of articles on depressive and anxiety symptoms.

From a total of 418 records identified through database searching, 167 items were screened by title and abstract, and 41 items were assessed in full text. After the exclusion of noneligible articles, 18 studies were found to fulfill the eligibility criteria and were included in the review. For a flow diagram summarizing the article selection process, see Figure 1.

#### 2.4.2. Quality of life

**Figure 2:**
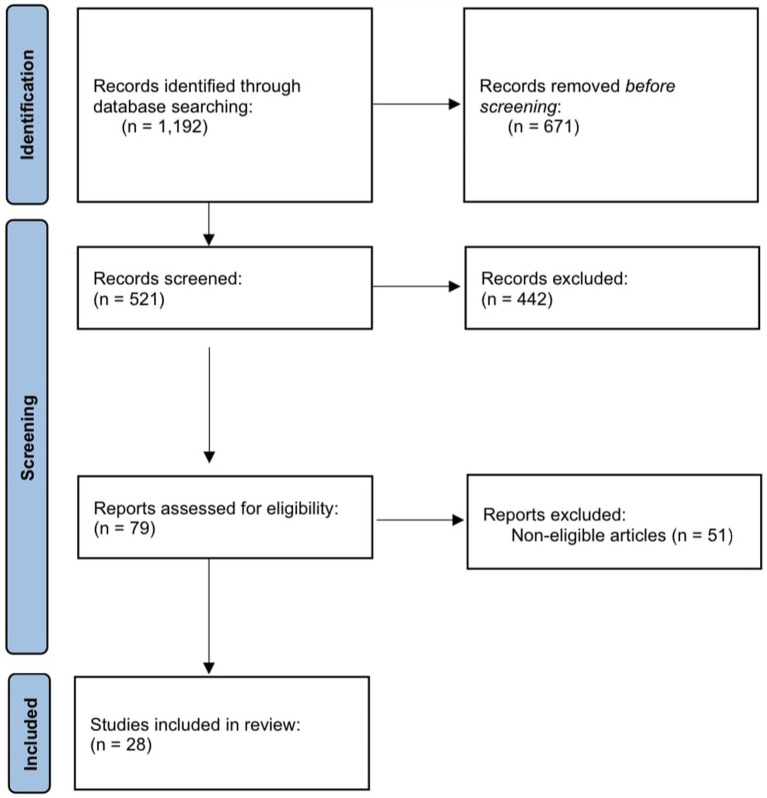
Flow diagram summarizing the selection process of articles on quality of life.

From a total of 1,192 records identified through database searching, 521 items were screened by title and abstract, and 79 items were assessed in full text. After the exclusion of noneligible articles, 28 studies were found to fulfill the eligibility criteria and were included in the review. For a flow diagram summarizing the article selection process, see Figure 2.

## 3. Results

### 3.1. Depressive and anxiety symptoms

#### 3.1.1. Study characteristics

A total of 18 studies met the inclusion criteria and were included in the review. The general characteristics of the articles included in the review and their main relevant findings are presented in Table 1. Most of the articles we published during the last decade. The number of participants per study ranged from 23 to 219, 928 (total *n* = 288,374). The total mean age of the population was 32.45 years, and in two studies, the information was not given. The diagnostic criteria for endometriosis included surgical (55.6%), histological (27.8%), clinical (33.3%), and registered diagnosis (11.1%); self-report (11.1%); or imaging techniques such as ultrasonography or magnetic resonance (27.8%).

**Table 1:** Characteristics and outcomes of the included studies evaluating the impact of endometriosis on depressive and anxiety symptoms.

Study	Sample size, *n*	Mean age, years	Diagnosis of endometriosis	Evaluation of depressive or anxiety symptoms	Relevant findings
Cavaggioni et al. (13)	80	35.0	Surgical or histological	Clinical interview, questionnaire (SCL-90)	Higher rate of clinically recognized mood and anxiety disorders; higher levels associated with pelvic pain; and higher SCL-90 score for depression
Estes et al. (14)	219,928	34.2	Clinical or surgical	Clinical interview	Higher rate of clinically recognized anxiety and depression; higher levels associated with pain
Facchin et al. (15)	246	34.05	Surgical or registered diagnosis	Questionnaire (HADS)	Higher HADS-d and HADS-a scores for endometriosis and bad quality of sleep
Geller et al. (16)	247	31.3	Self-report	Questionnaire (PHQ-9) Questionnaire (GAD-7)	Higher prevalence of depression, highest levels in the endometriosis participants with other diseases; higher prevalence of anxiety
He et al. (17)	133	35.8	Histological	Questionnaire (HAMA), Questionnaire (SDS)	Higher prevalence of depression and anxiety
Lorençatto et al. (18)	100	34.05	Not given	Questionnaire (BDI)	Higher prevalence of depression, higher levels associated with chronic pelvic pain
Matasariu et al. (19)	205	35.46	Not given	Questionnaire (BDI)	Higher prevalence of severe depression in women with the associated infertility
Mousa et al. (20)	2,610	34.18	Surgical	Self-report	Higher prevalence of depression and anxiety
Muharam et al. (21)	160	Not given	Clinical, surgical, histopatological, or US	Clinical interview	Higher rate of clinically recognized anxiety and depression
Mundo-López et al. (22)	230	36.7	Surgical, MR/US or clinical	Questionnaire (HADS), Questionnaire (EVEA)	Higher prevalence of depression and anxiety, higher levels associated with chronic fatigue
Olliges et al. (23)	23	31.4	Histological	Questionnaire (HADS), Questionnaire (STAI)	Higher HADS-d and HADS-a scores for endometriosis; no significant group differences were found for state anxiety
Quiñones et al. (24)	67	30.4	Surgical or clinical	Questionnaire (STAI)	Higher STAI score for endometriosis for trait anxiety, negatively associated with cortisol levels
Ribeiro et al. (25)	40	Not given	US	Questionnaire (HADS)	Higher HADS-d and HADS-a scores for endometriosis; 77.1% of the patients exhibited anxiety and depression simultaneously
Robert et al. (26)	63, 160	38.3	Clinical	Clinical interview	Higher rate of clinically recognized anxiety and depression, associated with longer hospital length of stay and higher total charges
Rossi et al. (27)	349	31.6	Registered diagnosis or self-report	Registered diagnosis	Higher prevalence of mood disorders in women with endometriosis
Škegro et al. (28)	79	35.03	Surgical, histopathological	Questionnaire (DASS-21)	Higher prevalence of depression and anxiety, associated with worse health status and infertility
Sullivan-Myers et al. (29)	471	31.5	Surgical, clinical, MRI, or US	Questionnaire (DASS-21)	Prevalence of depression associated with body image and self-esteem
Warzecha et al. (30)	246	33.5	Surgical or US	Questionnaire (EPQ)	Higher prevalence of depression, positively correlated with the age of the onset of dyspareunia, associated with chronic pelvic pain and painful defecation, independent from the stage of the disease, infertility and the duration of treatment

The table displays for each included study the citation, sample size, mean age of participants, diagnosis, method of evaluation, and main relevant findings. MR, Magnetic resonance; US, Ultrasonography; SCL-90, Symptom checklist-90; HADS, Hospital anxiety and depression scale; PHQ-9, Patient health questionnaire; GAD-7, The generalized anxiety disorder scale; HAMA, Hamilton anxiety scale; SDS, Self-rating depression scale; BDI, Beck depression inventory; EVEA, Scale for mood assessment; DASS-21, Depression anxiety stress scales; EPQ, Endometriosis patient questionnaire.

#### 3.1.2. Assessment tools

The most commonly applied tool to evaluate the prevalence of depressive and anxiety syndromes was a structured questionnaire. With regard to the assessment of depressive symptoms, among the most common questionnaires used were the Hospital Anxiety and Depression Scale (22.2%) and Beck Depression Inventory (11.1%). Concerning the evaluation of anxiety symptoms, the most used questionnaire was the State and Trait Anxiety Inventory (11.1%). Three studies (16.7%) performed a structured clinical interview to assess depressive and anxiety symptoms, and one study (5.6%) based the assessment of the outcomes on a self-reporting measurement tool.

#### 3.1.3. Results

Depending on the assessment tool used, depressive symptoms occurred in 9.8–98.5% of patients with endometriosis and anxiety symptoms occurred in 11.5–87.5%. In comparison, depressive symptoms occurred in 6.6–9.3% of control groups in the included studies and anxiety symptoms in 6.0–10.1%. Estes et al. (14) reported an increased risk of developing clinically recognized depression (HR: 1.48, 95% CI: 1.44–1.53) and anxiety (HR: 1.38, 95% CI: 1.34–1.42) for patients with endometriosis compared with women never diagnosed with endometriosis. In the same study, it was observed that the hazard ratios for depression were stronger in women younger than 35 years than women ≥35 years of age.

A strong association was reported between higher rates of depression and anxiety symptoms and endometriosis-associated pain (i.e., chronic pelvic pain, dysmenorrhea, dyspareunia, or painful defecation) and pain-related comorbidities (13, 14, 18, 29). Warzecha et al. (30) observed that the prevalence of depression was positively correlated with the age of the onset of dyspareunia (14.5 years of age, SD = 4.3 vs. 19.6, SD = 7.4 in the group without depression, *p* = 0.002). Inconclusive findings were reported in regard to infertility. Two studies found that higher levels of depression were associated with infertility (19, 30), and one study reported that the diagnosis of infertility was not related to the incidence of depression (OR = 0.7 95% CI 0.4–1.4) (29). Higher rates of depression and anxiety were associated with fatigue (14, 28). One study found that the incidence of depressive symptoms or chronic fatigue was independent of the stage of endometriosis (*p* = 0.8 to 0.9 for each stage) (29). The same study reported that the duration of treatment of endometriosis was not related to the incidence of depression (OR = 0.9 95% CI 0.8–1.2).

Other risk factors associated with incident depression and incident anxiety included prior use of opioid analgesics and asthma. Prior use of gonadotropin-releasing hormone agonists and oral contraceptives was associated with elevated rates of depression, and interstitial cystitis, allergic rhinitis, and allergies were associated with elevated rates of anxiety (14). One study reported an association between body image disturbances and the negative response of depression symptoms (18). Several factors were associated with significantly lower rates of depression and anxiety among women with endometriosis. Women who had a prior pregnancy, uterine fibroids, and hyperlipidemia had lower rates of depression and anxiety. Vitamin D deficiency was associated with a low risk of depression, and the use of oral contraceptives was associated with a low risk of anxiety (14).

Robert et al. (26) observed that women with psychiatric comorbidities such as depression and anxiety had a longer hospital stay and higher total charges compared with the non-psychiatric cohort.

### 3.2. Quality of life

#### 3.2.1. Study characteristics

A total of 28 studies met the inclusion criteria and were included in the review. The general characteristics of the articles included in the review and their main relevant findings are presented in Table 2. Most of the articles were published during the last decade. The number of participants per study ranged from 12 to 2,004 (total *n* = 6,578). The total mean age of the population was 34.7 years, and in three studies, the information was not given. The diagnostic criteria for endometriosis included surgical (60.7%), histological (28.6%), clinical (28.6%), self-report (10.7%), or imaging techniques such as ultrasonography or magnetic resonance (28.6%).

**Table 2:** Characteristics and outcomes of the included studies evaluating the impact of endometriosis on QoL.

Study	Sample size, *n*	Mean age, years	Diagnosis of endometriosis	Evaluation of QoL	Relevant findings
Abd El-Kader et al. (31)	109	31.9	Surgical	GQOL	Impaired HRQoL scores associated with adhesions related to endometriosis
Adoamnei et al. (32)	99	36.3	Clinical, US	SF-12	Impaired HRQoL scores in patients with endometriosis compared to controls
Álvarez-Salvago et al. (33)	25	36.2	Clinical	SF-12	Impaired HRQoL scores in regard to physical domain compared to controls; no significant differences between groups in the mental domain were found
Bień et al. (34)	309	30.86	Surgical, histological	WHOQOL-BREF	Impaired HRQoL scores associated with their acceptance of illness, BMI, negative impact of symptoms on the relationship with the partner, and dyspareunia
Chen et al. (35)	371	36.4	Histological	SF-12	Higher HRQoL scores in the Chinese patients in comparison with the Italian patients; quality of life of endometriotic patients associated with ethnicity
Chmaj-Wierzchowska et al. (36)	23	29.83	Surgical	NHP	Impaired HRQoL scores in patients with endometriosis compared to controls
de Freitas Fonseca et al. (37)	77	Range 21–46	Clinical, MRI, surgical, and histological	SF-36, EHP-30	Impaired HRQoL scores associated with dysmenorrhea and chronic pelvic pain
Facchin et al. (15)	123	34.05	Surgical, clinical	SF-12	Impaired HRQoL scores associated with sleep disturbances
Florentino et al. (38)	65	39.7	US, surgical	EHP-30	Impaired HRQoL scores associated with dyspareunia and acyclic pain
Fourquet et al. (39)	193	33.2	Self-report	SF-12, EHP-5	Impaired HRQoL scores associated with CPP and infertility
González-Echevarría et al. (40)	24	Range 13–25	Surgical	EHP-5	The QOL of patients with endometriosis is negatively correlated with anxiety, depression and maladaptive coping strategies, but not pain levels
He et al. (17)	139	35.8	Histological	SF-12	The QOL of patients with endometriosis is negatively correlated with anxiety and depression
Hernández et al. (41)	112	35.5	Clinical, US, surgical, and histological	SF-12	Impaired HRQoL scores in patients with deep endometriosis compared to controls; The QOL of patients with endometriosis in regard to physical domain is positively correlated with age; no such correlation in the mental domain was found
Matasariu et al. (19)	205	35.46	Not given	EHP-30	Impaired HRQoL scores associated with lack of control in patients with endometriosis-caused infertility
Mousa et al. (20)	518	34.04	Surgical	SF-36	Impaired HRQoL scores associated with susceptibility and severity of multiple pain syndromes and infertility
Muharam et al. (21)	160	Not given	Clinical, US, surgical, and histopatological	EHP-30	Lower HRQoL associated with severe endometriosis-related pain and depression
Mundo-López et al. (22)	230	36.4	Clinical	EHP-30	Lower HRQoL associated with fatigue
Nnoaham et al. (42)	745	32.5	Surgical	SF-36	Impaired HRQoL scores in women with endometriosis when compared with those with similar symptoms and no endometriosis; impaired HRQoL associated with status of employment, pain, and longer diagnostic delays
Olliges et al. (23)	12	33.67	Histological	SF-12	HRQoL impaired in women with endometriosis; lower SF-12 scores in regard to mental and physical QoL
de Farias Rodrigues et al. (43)	106	34.66	Surgical	SF-36	Impaired HRQoL scores associated with clinical manifestations, such as dyspareunia and pain, but not the degree of endometriosis
Škegro et al. (28)	79	35.03	Surgical, histopathological	EHP-5	The QOL of patients with endometriosis is negatively correlated with pain and depressive symptoms
Soliman et al. (44)	2,004	35.5	Self-report	SF-12, EHP-30	The QOL of patients with endometriosis is negatively correlated with endometriosis symptom severity
Souza et al. (45)	57	35.8	Surgical	WHOQOL-BREF	Lower HRQoL associated with CPP; similar QoL scores in women with and without endometriosis suffering from chronic pelvic pain
Thammasiri et al. (46)	99	35.2	US	EHP-30	Lower HRQoL associated with CPP
Touboul et al. (47)	159	34.8	Clinical, MRI	EQ-5D	HRQoL impaired in women with deep infiltrating endometriosis; reduced HRQoL associated with dysmenorrhea, dyspareunia, CPP, diarrhea, constipation, and painful defecation, but not the infertility or patient’s age
Van Niekerk et al. (48)	318	30.8	Self-reported, surgical	SF-36	HRQoL impaired in women with endometriosis; higher HRQoL scores associated with higher levels of self-compassion and body compassion
Verket et al. (49)	157	35.2	Surgical	SF-36	HRQoL impaired in women with moderate to severe endometriosis; poorer mental HRQoL compared with women with rheumatoid arthritis; and reduced HRQoL associated with pain and the combination of infertility and childlessness
Yela et al. (50)	60	37.7	Surgical, US, and MR	SF-36, EHP-30	HRQoL impaired in women with deep endometriosis, regardless of the questionnaire used for evaluation

The table displays for each included study the citation, sample size, mean age of participants, diagnosis, method of evaluation, and main relevant findings. US, Ultrasonography; MR, Magnetic resonance; GQOL, Global quality of life scale; SF, short-form health survey; WHOQOL-BREF, World Health Organization Quality of Life-Br f; NHP, Nottingham health profile questionnaire; EHP, Endometriosis health profile; EQ-5D, European quality of life-5 dimensions; EPQ, Endometriosis patient questionnaire; and CPP, chronic pelvic pain.

#### 3.2.2. Assessment tools

From the 28 selected publications, a total of eight scales for evaluation of quality of life in patients with endometriosis were identified. Thirteen of the included studies applied generic scales, and 11 studies used endometriosis-specific instruments. The most frequently used instruments were SF-36 (25%) and its short form the SF-12 (32.1%) for the generic questionnaires and EHP-30 (28.6%) and its short form the EHP-5 (10.7%) for the specific ones. Both generic and disease-specific instruments were used to evaluate the quality of life in endometriosis in four of the included studies, producing similar results (26, 37, 39, 44). Generic scales allow comparisons between patients with endometriosis and the general population. Disease-specific questionnaires better reflect the many aspects and properties of HRQoL that are important to women with endometriosis (50).

#### 3.2.3. Results

The results showed that the quality of life in patients with endometriosis was significantly impaired, regardless of the tool used for evaluation. The impairment of QoL in women affected by endometriosis is greater in both mental and physical spheres when compared to non-endometriosis conditions that appear to not affect the physical sphere of QoL (4). Lower HRQoL scores were found in comparison with women from the general population (23, 32, 33, 36, 42, 51), and poorer mental health was observed when compared with women with rheumatoid arthritis (49). Inconsistent findings on the impairment of HRQoL were observed in comparison with those with similar symptoms and no endometriosis. Nnoaham et al. (42) reported a significantly reduced physical HRQoL in women with endometriosis (*n* = 745) compared with symptomatic women without endometriosis (*n* = 587). However, Souza et al. (45) with a smaller sample size found that the presence of endometriosis in addition to chronic pelvic pain was not an independent factor impacting quality of life.

HRQoL was diminished in the large majority of the health scales of the SF-12 (seven out of eight) in Spanish women with endometriosis when compared with the control group, suggesting the influence of the disease on patients’ quality of life (22). The same study found that women with endometriosis were 8.36 times (95% CI: 4.06–17.2) more likely than controls to have lower scores in the physical domain of quality of life. Six other studies found significantly reduced physical HRQoL in affected women (32, 36, 37, 39, 42, 45). Inconsistent findings were reported in regard to the mental health component of HRQoL. Three studies noted disability in mental health status in patients with endometriosis (37, 39, 42), two studies found no significant differences between endometrial and control groups (32, 51), and one study reported higher mental health scores than the reference population (45). It was also reported that endometriosis had a negative impact on work productivity across countries and ethnicities, mainly owing to reduced effectiveness at work (36, 37).

Inconsistent findings on the quality of life in women with endometriosis were observed regarding its correlation with age. One study reported a positive correlation between the impairment of HRQoL and age (31). However, no such correlation was found in another study (41). Higher HRQoL scores were associated with higher education levels (20) and status of employment (36). Both the physical and mental scores of women in the Chinese population were higher than those of women in the Italian population, suggesting that ethnicity may affect quality of life in patients with endometriosis (35).

Quality of life was significantly impaired by the presence of adhesions resulting from endometriosis when compared with endometrial women without adhesions (49). No statistically significant differences were found in QoL based on the degree of endometriosis (45). A negative correlation between the QoL scores of the patients and their BMI was found (34). Pain was the main symptom correlated with impaired QoL in women with endometriosis (19, 20, 23, 30, 31, 36, 39, 43). Poor HRQoL was associated with different types of endometriosis pain, such as dysmenorrhea (26, 34, 47), dyspareunia (21, 31, 34, 47), acyclic pain (21, 39), and chronic pelvic pain (26, 34, 37, 38, 49). In addition, QoL was negatively correlated with pain intensity (26, 39, 47, 49). Another indicator of lower HRQoL was the presence of dyschezia (painful defecation) and diarrhea or constipation (34). In regard to infertility, inconsistent findings were reported. A negative relationship between endometriosis and infertility was found in three studies (19, 37, 43), and similar HRQoL in infertile and non-infertile women with endometriosis was found in two studies (23, 34). However, Verket et al. (49) found that childless infertile women had significantly lower mental HRQoL in comparison with infertile women with children, suggesting it may not be infertility *per se* but the combination of infertility and childlessness that affects mental health in women with endometriosis.

The HRQoL of patients with endometriosis was negatively correlated with anxiety and depression (19, 40, 43, 46). Facchin et al. (15) reported a strong association between sleep disturbances and poorer psychological health and quality of life in women with endometriosis. In addition, a strong association between fatigue and lower QoL was found (28). Other independent correlation factors in relation to HRQoL were the acceptance of illness, the impact of symptoms on the relationship with the partner (47), body compassion and self-compassion (48), the loss of control over the symptoms and altered emotional status (30), and maladaptive coping strategies (46).

Nnoaham et al. (42) reported an association between longer diagnostic delay and reduced physical HRQoL in affected women.

## 4. Discussion

Endometriosis is a disease that affects both the physical and psychological health of patients. The aim of this systematic review was to give an overview of the prevalence of depressive and anxiety symptoms and quality of life assessment in women with endometriosis. We also investigated the dependent factors correlated with those associations. Each of the studies included in the review reported a high rate of prevalence of depressive and anxiety symptoms in women with endometriosis. Ribeiro et al. (25) reported that 77.1% of patients exhibited anxiety and depression simultaneously, indicating a high rate of comorbidity in women with endometriosis. In addition, it was reported that patients with endometriosis had an increased risk of developing clinically recognized depression and anxiety compared with women never diagnosed with endometriosis. The evidence presented should raise awareness of the link between endometriosis and the psychological functioning of the patients. We suggest integrating a psychological assessment of women with endometriosis in order to identify those at risk of developing symptoms of depression or anxiety and providing them with adequate support to improve the psychological outcomes.

The quality of life of patients with endometriosis was significantly impaired. The HRQoL scores were lower in comparison with women from the general population and women with rheumatoid arthritis, but inconsistent findings were reported when compared with those with similar symptoms and no endometriosis. More studies are needed to assess the impairment of HRQoL in conditions representing chronic pelvic pain to further evaluate this association.

The studies investigating sociodemographic characteristics associated with higher rates of affective symptoms found that women with endometriosis younger than 35 years of age had a higher rate of clinically recognized depression. Inconsistent findings were reported on the correlation between age and quality of life in patients with endometriosis, whereas a positive association was found between higher education levels or status of employment and HRQoL.

It has been reported that it is not the stage of endometriosis that impacts the QoL of women with endometriosis, but rather the clinical manifestations of the disease (34). Endometriosis-associated pain such as chronic pelvic pain, dysmenorrhea, dyspareunia, or painful defecation was associated with a higher prevalence of depression and anxiety symptoms and lower QoL. The incidence of depressive and anxiety symptoms appears to be higher in endometriosis patients compared with other forms of chronic pelvic pain (4). Pain intensity was positively correlated with depressive and anxiety scores, and a negative correlation was found between pain intensity and HRQoL scores, which led to the interpretation that the more intense the pain, the higher the occurrence of affective symptoms and the worse the quality of life. The current evidence does not allow for the conclusion that infertility in women with endometriosis is associated with higher levels of depression and anxiety or impairment of QoL. Perhaps it is the combination of infertility and childlessness that affects the mental health of women with endometriosis, as Verket et al. (49) found that childless infertile women had significantly lower mental HRQoL in comparison with infertile women with children. Studies investigating quality of sleep as a dependent factor reported a strong association between sleep disturbances and poorer psychological health and quality of life in women with endometriosis. In addition, bad quality of sleep among endometriosis patients was associated with greater fatigue, and fatigue was correlated with higher rates of depression and anxiety and lower QoL scores. Those findings suggest a need for a holistic approach in healthcare assistance for endometriosis patients. A better integration of all aspects of patient care is likely to improve both the physical and psychological outcomes.

Other studies included in this review suggest that risk factors correlated with depressive and anxiety symptoms in women with endometriosis include prior use of opioid analgesics and asthma. Elevated rates of depression were associated with prior use of gonadotropin-releasing hormone agonists and oral contraceptives; and interstitial cystitis, allergic rhinitis, and allergies were associated with elevated rates of anxiety. Independent correlation factors in relation to HRQoL include the acceptance of illness, the impact of symptoms on the relationship with the partner, the loss of control over the symptoms and altered emotional status, and maladaptive coping strategies. These findings indicate that the relationship between endometriosis and poorer psychological health and impaired quality of life is complex and that many dependent factors are involved in this association. Awareness of the relationship between endometriosis and mental health of the patients informs tailored care and allows for a patient-centered approach.

Furthermore, an association between longer diagnostic delay and reduced HRQoL was reported, which indicates the importance of heightened awareness of the disease among clinicians. Psychiatric comorbidities such as depression and anxiety in women with endometriosis are associated with a longer hospital mean length of stay and higher total charges. In addition, endometriosis was found to exert a negative impact on productivity at work. Earlier diagnosis and symptom control strategies should translate into improving psychological health, a shorter hospital length of stay, lower total charges, and better work productivity of the affected women.

## 5. Conclusion

This systematic review shows that endometriosis is associated with depressive and anxiety symptoms and impaired HRQoL. Broad correlating factors modulate mental health outcomes, indicating a complex relationship between the disease and the psychological health of the patients. We hope that the evidence presented in this paper will raise awareness of the link between endometriosis and symptoms of depression and anxiety. We suggest integrating a psychological assessment of these patients in order to identify those at risk of developing mental health issues and providing them with adequate support. A better integration of all aspects of patient care is likely to improve both the physical and psychological outcomes.

## Data availability statement

The original contributions presented in the study are included in the article/supplementary material, further inquiries can be directed to the corresponding author.

## Author contributions

MS and RT contributed to conception and design of the study. MS, RT, and KK contributed to the methodology of the study and reviewed and edited the sections of the manuscript. RT performed the formal analysis. MS and KK organized the database and curated the data. MS wrote the first draft of the manuscript. RT and KK were responsible for the project administration. All authors contributed to the article and approved the submitted version.

## Conflict of interest

The authors declare that the research was conducted in the absence of any commercial or financial relationships that could be construed as a potential conflict of interest.

## Publisher’s note

All claims expressed in this article are solely those of the authors and do not necessarily represent those of their affiliated organizations, or those of the publisher, the editors and the reviewers. Any product that may be evaluated in this article, or claim that may be made by its manufacturer, is not guaranteed or endorsed by the publisher.
